# Comparison of Multiparametric Magnetic Resonance Imaging with Prostate-Specific Membrane Antigen Positron-Emission Tomography Imaging in Primary Prostate Cancer Diagnosis: A Systematic Review and Meta-Analysis

**DOI:** 10.3390/cancers14143497

**Published:** 2022-07-19

**Authors:** Yi Zhao, Benjamin S. Simpson, Naomi Morka, Alex Freeman, Alex Kirkham, Daniel Kelly, Hayley C. Whitaker, Mark Emberton, Joseph M. Norris

**Affiliations:** 1 School of Medicine, Imperial College London, London SW7 2BX, UK; 2 UCL Cancer Institute, University College London, London WC1E 6BT, UK; b.simpson@ucl.ac.uk; 3 UCL Medical School, University College London, London WC1E 6BT, UK; naomi.morka.17@ucl.ac.uk; 4 Department of Pathology, University College London Hospitals NHS Foundation Trust, London NW1 2PG, UK; alex.freeman2@nhs.net; 5 Department of Radiology, University College London Hospitals NHS Foundation Trust, London NW1 2PG, UK; alexkirkham@nhs.net; 6 School of Healthcare Sciences, Cardiff University, Cardiff CF10 3AT, UK; kellydm@cardiff.ac.uk; 7 UCL Division of Surgery & Interventional Science, University College London, London WC1E 6BT, UK; hayley.whitaker@ucl.ac.uk (H.C.W.); m.emberton@ucl.ac.uk (M.E.); joseph.norris@ucl.ac.uk (J.M.N.); 8 Department of Urology, University College London Hospitals NHS Foundation Trust, London NW1 2PG, UK

**Keywords:** prostate-specific membrane antigen positron-emission tomography, multiparametric magnetic-resonance imaging, primary diagnosis, prostate cancer, systematic review

## Abstract

**Simple Summary:**

Multiparametric magnetic-resonance imaging (mpMRI) is a routinely used imaging modality for diagnosing prostate cancer but misses 10–20% of prostate tumours. Recently, prostate-specific membrane antigen positron-emission tomography (PSMA PET) has been proposed as an alternative to mpMRI for diagnosis. Our systematic review and meta-analysis aimed to compare the diagnostic performance between mpMRI and PSMA PET modalities prior to biopsy. Ten articles directly comparing the performance of both modalities in the same patient cohort were investigated. PSMA PET/CT was superior in diagnosing patients with prostate cancer over mpMRI, but not in defining the location of the cancer. Early evidence suggests that the addition of PSMA PET within the diagnostic pathway may enhance the detection of clinically significant prostate cancer.

**Abstract:**

Multiparametric magnetic-resonance imaging (mpMRI) has proven utility in diagnosing primary prostate cancer. However, the diagnostic potential of prostate-specific membrane antigen positron-emission tomography (PSMA PET) has yet to be established. This study aims to systematically review the current literature comparing the diagnostic performance of mpMRI and PSMA PET imaging to diagnose primary prostate cancer. A systematic literature search was performed up to December 2021. Quality analyses were conducted using the QUADAS-2 tool. The reference standard was whole-mount prostatectomy or prostate biopsy. Statistical analysis involved the pooling of the reported diagnostic performances of each modality, and differences in per-patient and per-lesion analysis were compared using a Fisher’s exact test. Ten articles were included in the meta-analysis. At a per-patient level, the pooled values of sensitivity, specificity, and area under the curve (AUC) for mpMRI and PSMA PET/CT were 0.87 (95% CI: 0.83–0.91) vs. 0.93 (95% CI: 0.90–0.96, *p* < 0.01); 0.47 (95% CI: 0.23–0.71) vs. 0.54 (95% CI: 0.23–0.84, *p* > 0.05); and 0.84 vs. 0.91, respectively. At a per-lesion level, the pooled sensitivity, specificity, and AUC value for mpMRI and PSMA PET/CT were lower, at 0.63 (95% CI: 0.52–0.74) vs. 0.79 (95% CI: 0.62–0.92, *p* < 0.001); 0.88 (95% CI: 0.81–0.95) vs. 0.71 (95% CI: 0.47–0.90, *p* < 0.05); and 0.83 vs. 0.84, respectively. High heterogeneity was observed between studies. PSMA PET/CT may better confirm the presence of prostate cancer than mpMRI. However, both modalities appear comparable in determining the localisation of the lesions.

## 1. Introduction

The introduction of multiparametric magnetic-resonance imaging (mpMRI) has improved the diagnostic pathway for suspected prostate cancer (PCa) [1]. Recently, a novel imaging modality, prostate-specific membrane antigen emission tomography (PSMA PET), has demonstrated potential as an adjunctive or alternative imaging technique for primary prostate cancer diagnosis [2]. PSMA, a type 2 transmembrane glycoprotein, is known to be overexpressed in prostate tumours [3,4], and its level of expression correlates with high serum levels of prostate-specific antigen (PSA) and a higher Gleason score. As such, PSMA may provide greater utility as a more targeted and specific marker of prostate cancer [5].

Although mpMRI is now commonly used in the diagnosis process, previous studies showed that around 10–20% of missed diagnoses are clinically significant prostate tumours [6,7]. In addition, PSMA PET has demonstrated efficacy as a useful staging tool for prostate cancer and for detecting metastases [5,8].

It is unclear whether PSMA PET may offer an improved ability to diagnose primary prostate cancer over mpMRI and whether it has sufficient sensitivity to pinpoint tumour location. In this study, we systematically reviewed the evidence comparing the diagnostic accuracies of mpMRI and PSMA-PET for detecting clinically significant diseases. The comparators tested between the two imaging modalities included sensitivity, specificity, and overall AUC values for both the presence of PCa (patient-level) and location of the lesion (lesion-level).

## 2. Evidence Acquisition

### 2.1. Study Design

This review was prospectively registered with the PROSPERO International Registry (CRD42021239296). The protocol for this systematic review and meta-analysis has been published previously and was based on the Preferred Reporting Items for Systematic Review and Meta-Analysis Protocols (PRISMA-P) statement [9].

### 2.2. Literature Search

A systematic literature search was conducted across four databases—MEDLINE, PubMed, EMBASE, and Cochrane—to retrieve all relevant studies. Controlled Medical Subject Heading (MeSH) terms were selected to refine the relevance of studies and reduce the number of unrelated studies. Multiple synonyms of the term “mpMRI” and “PSMA PET” were used in the search strategy to account for variations in terminology. The final search strategy contained 17 components linked by AND/OR operator terms: Prostat* AND (Cancer OR Tumo* OR malignan* OR adenocarcinoma OR lesion* OR Disease) AND (PSMA OR “prostate-specific membrane antigen positron emission tomography”) AND (MR OR magnetic resonance imaging OR MP-MRI OR multiparametric MRI OR multiparametric magnetic resonance imaging OR multiparametric MRI OR “multiparametric magnetic resonance imaging”) AND Diagnosis.

### 2.3. Study Selection

**Figure 1:**
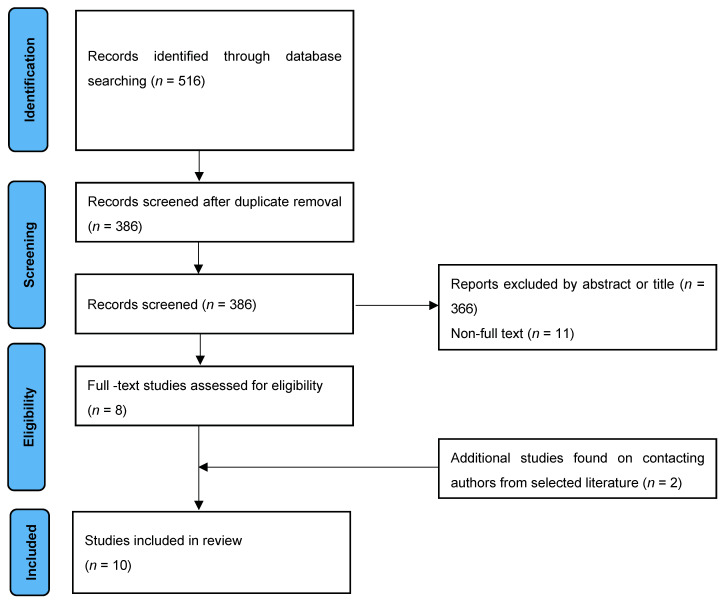
PRISMA flow diagram of evidence acquisition. PRISMA—Preferred Reporting Items for Systematic Reviews and Meta-Analysis.

All retrieved studies published between July 1977 to December 2021 were uploaded to Rayyan, a semi-automated tool to assist in the further selection of articles efficiently and accurately [10]. Figure 1 illustrates an overview of the study selection process. In order to be included, studies had to compare the diagnostic accuracies of PSMA PET and mpMRI for the primary diagnosis of prostate cancer. Studies of interest were those comparing the sensitivity and specificity of both modalities separately. The reference standard for histopathology was whole-mount prostatectomy or prostate biopsy. Expert opinions, correspondence articles, conference abstracts, review articles, and case reports were excluded. Any studies that were not written in the English language were also excluded. Included studies made a direct comparison between PSMA PET and mpMRI. Articles that focused on investigating the combined accuracy of both modalities or solely on the diagnostic accuracy of PSMA PET or mpMRI alone were also removed.

### 2.4. Data Collection

All extracted data were collected using a standardised form and were checked independently by each reviewer. The data collection types included the following: the year of publication and the study authors, the study design and the patient demographics, the specification concerning the methodology, and the reported number of true/false positives and true/false negatives [9]. Three investigators (Y.Z., J.M.N. and B.S.S.) independently screened all eligible studies, assessing both the titles and abstracts for relevance. Reference sections of included articles were also manually searched to identify missed studies and additional data. Full-text articles were then retrieved for further review of eligibility.

### 2.5. Quality Assessment

Risk-of-bias assessment was conducted using the QUADAS-2 score [11]. The description of this method has also been described in previous systematic review articles [12]. Scoring of the QUADAS-2 score is split into four main domains: patient selection, index test, reference standard, flow, and timing. This bias assessment was conducted to assess the applicability and reliability of the data produced. Studies with low quality or suggesting a high level of bias were excluded or included with appropriate commentary [13].

### 2.6. Data Synthesis

Primarily, our endpoint was statistically significant differences in quantitative measurements such as sensitivity, specificity, PPV, and NPV in determining diagnostic accuracies between PSMA PET and mpMRI. An additional focus was to derive critical themes within the retrieved literature, such as the utility of different mpMRI scoring systems, including the Prostate Imaging-Reporting and Data System (PI-RADS), Likert score, and other radiogenomic features, as well as the criteria used to define clinically significant prostate cancer (csPCa), including PI-RADS or Likert score thresholds.

### 2.7. Meta-Analysis

The individual study’s true positives (TPs), false negatives (FNs), true negatives (TNs), and false positives (FPs) were extracted to build a 2 × 2 contingency table based on the detection of csPCa via mpMRI and PSMA PET/CT. Pooled quantitative sensitivities and specificities were compared using bivariate analysis, with 95% confidence intervals (CIs) presented. The summary receiver operating characteristic (SROC) curves were then generated using the area-under-the-curve (AUC) values presented. Normality was assessed using density plots for the distribution of untransformed, logit, and double-arcsine-transformed proportions and confirmed using a Shapiro–Wilk test. The set of values most resembling a normal distribution was used in the combined analysis. Heterogeneity and inter-study variation were quantified through I^2^, and a random-effects model was applied for estimation with partial pooling. Leave-one-out analysis was performed to detect potential outliers, and studies with a statistically significant influence on the fitted model were removed and the model re-fitted. Summary comparisons between PSMA PET and mpMRI were estimated once heterogeneity had been minimised through outlier removal. A Fisher’s exact test was conducted to assess statistically significant differences between the two diagnostic tests, with *p* < 0.05 considered statistically significant. All data analysis and visualisation were performed in the R statistical environment (version 4.1.1, 10 August 2021) using the “mada” and “meta” packages.

## 3. Evidence Synthesis

### 3.1. Study Characteristics

Overall, 516 articles were retrieved: 135 from EMBASE, 71 from Medline, 373 from PubMed, and none from Cochrane. From these studies, ten articles were eligible for further analysis (Table 1) [14,15,16,17,18,19,20,21,22,23]. The included studies were published between 2016 and 2021. A total of 918 patients and 540 lesions were included for intra-individual comparison between mpMRI and PSMA PET/CT imaging. All studies used 3.0 Tesla for MRI imaging, and two studies used both 1.5 Tesla power and 3.0 Tesla power in mpMRI imaging [15,19]. One study used the PI-RADS scoring system version 1.0 [22], while eight studies adopted PI-RADS v2 [14,15,16,17,18,19,20,21], and one study adopted the newest PI-RADS v2.1 [23]. For mpMRI, a lesion with a PI-RADS score > 3 was considered highly indicative of clinically significant prostate cancer in nine studies [14,15,17,18,19,20,21,22,23]. One study used a PI-RADS score > 4 as the threshold for clinically significant prostate cancer [16]. All but two studies used a 68 Ga-PSMA-11 tracer (HBED-CC), with one study using an 18 F-PSMA-1007 tracer and one using 68 Ga-PSMA-617 [21,23]. The range of the PSMA tracer injected was between 131.7 and 310 MBq in all studies. PSMA PET images were interpreted visually where regions of interest were compared with background uptake in all studies. A high suspicion of clinically significant cancer was defined using a 3- or 4-point Likert scale [17,18,19], based on the SUVmax value [14] or higher update to the background activity [15,21,22,23]. A score of equivocal and above, or probably positive and above, was considered a clinically significant cancer [18,19]. The histopathological definition of csPCa was based on a Gleason score ≥ 7 (3 + 4 or 4 + 3) [16,17,18,21,22,23] or the International Society of Urological Pathology (ISUP) grading [14,19,20], with three studies incorporating the tumour size in their csPCa definition [15,16,22]. The age of the included patients ranged from 62–69 years old. The range of mean PSA values in the included studies was 5.6–17.4 ng/dL (Table 1). Four articles were identified for PSMA PET/MRI analysis, but were not considered for meta-analysis due to the different definitions of PSMA PET/MRI [16,24,25,26].

### 3.2. Meta-Analysis

Sensitivity and specificity for both mpMRI and PSMA PET/CT were reported separately at per-patient and per-lesion levels. In the per-patient-level analysis, each case was regarded as an individual patient receiving both imaging modalities. In the per-lesion-level analysis, each case was regarded as an individual lesion identified in each histopathological sample.

#### 3.2.1. Per-Patient Analysis

**Figure 2:**
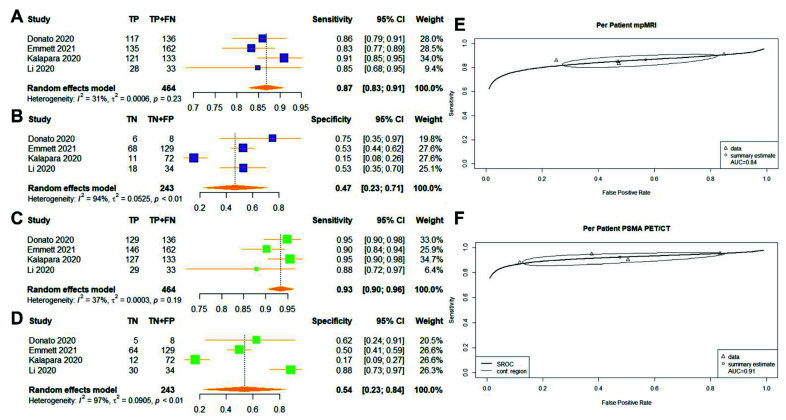
(**A**–**F**) Reported sensitivity and specificity values for both mpMRI and PSMA PET/CT with AUC values on SROC curves for per-patient analysis. Forest plots for pooled sensitivities and specificities are displayed in bold and as diamonds in the graphs for mpMRI (**A**,**B**) and PSMA PET/CT (**C**,**D**). The SROC curves indicate the summary estimates in circles (**E** for mpMRI; **F**, PSMA PET/CT). Triangles represent included study, with dotted lines representing the confidence interval and solid lines for the SROCs. AUC values are displayed in the legend. mpMRI = multiparametric magnetic-resonance imaging; PSMA PET/CT—prostate-specific membrane antigen positron-emission tomography/computed tomography; AUC—area under the curve, SROC—summary receiver operating characteristic.

Four studies were included in the paired analysis between mpMRI and PSMA PET/CT (Figure 2) [18,19,20,21]. A total of 707 patients were included, with 464 patients having proven csPCa. The pooled sensitivity for mpMRI and PSMA PET/CT was 0.87 (95% CI: 0.83–0.91) vs. 0.93 (95% CI: 0.90–0.96, *p* = 0.001657), and the pooled specificity was 0.47 (95% CI: 0.23–0.71) vs. 0.54 (95% CI: 0.23–0.84, *p* = 0.5225), respectively (Figure 2). The AUC values were 0.84 vs. 0.91, respectively (Figure 2). The heterogeneity between studies was large for specificity analysis for mpMRI (I^2^ = 0.94) and PSMA PET/CT (I^2^ = 0.97), with statically significant Cochrane Q statistics *p* < 0.01.

#### 3.2.2. Per-Lesion Analysis

**Figure 3:**
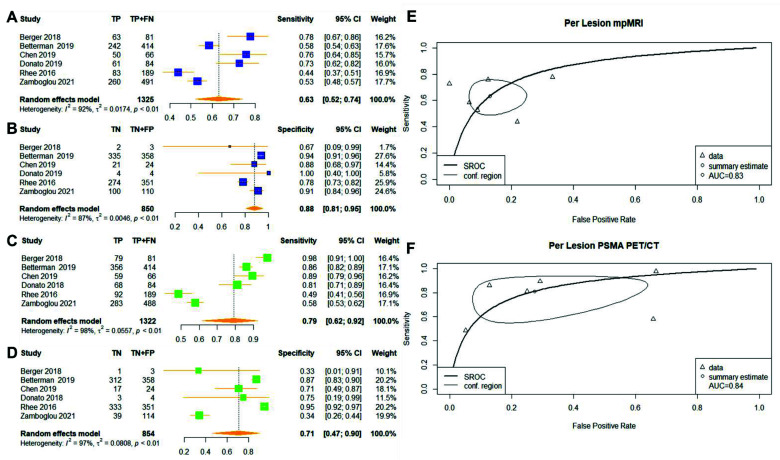
(**A**–**F**) Reported sensitivity and specificity values for both mpMRI and PSMA PET/CT with AUC values on SROC curves for per-lesion analysis. Forest plots for pooled sensitivities and specificities are displayed in bold and as diamonds in the graphs for mpMRI (**A**,**B**) and PSMA PET/CT (**C**,**D**). The SROC curves indicate the summary estimates in circles (**E** for mpMRI; **F**, PSMA PET/CT). Triangles represent included study, with dotted lines representing the confidence interval and solid lines for the SROCs. AUC values are displayed in the legend. mpMRI—multiparametric magnetic-resonance imaging; PSMA PET/CT—prostate-specific membrane antigen positron-emission tomography/computed tomography; AUC—area under the curve; SROC—summary receiver operating characteristic.

Six studies investigated the diagnostic accuracy of mpMRI and PSMA PET/CT at a lesion level (Figure 3) [14,15,16,17,22,23]. A ‘’lesion’’ was defined as individual tissue slices analysed by both imaging modalities with histopathological confirmation of the lesion location in the included studies. Of the 2175 lesions included in the analysis from 211 patients, 1325 were considered csPCa (Table 1). The pooled sensitivity values of mpMRI and PSMA PET/CT were lower at 0.63 (95% CI: 0.52–0.74) vs. 0.79 (95% CI: 0.62–0.92, *p* = 1.848 × 10^−12^), and the pooled specificity values were 0.88 (95% CI: 0.81–0.95) vs. 0.71 (95% CI: 0.47–0.90, *p* = 0.0226), respectively (Figure 3). The AUC values were 0.83 vs. 0.84, respectively (Figure 3). Heterogeneity remained large for the pooled sensitivity of mpMRI and for both the sensitivity and specificity of PSMA PET/CT, with statically significant Cochrane Q statistics *p* < 0.01.

### 3.3. Risk of Bias

**Figure 4:**
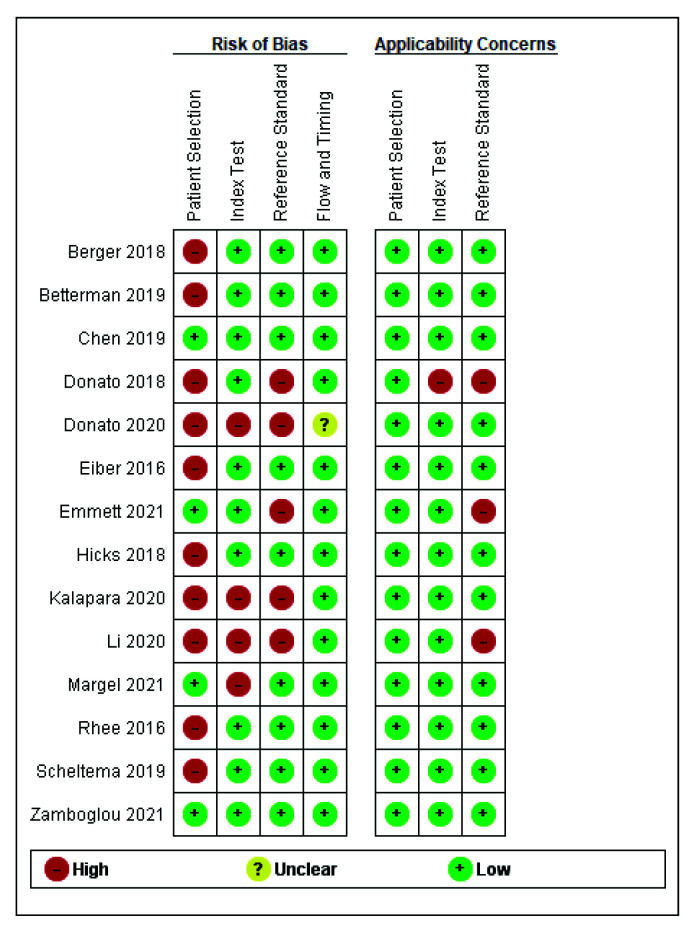
QUADAS-2 score indicates the risk of bias analysis in assessing the low, high, or unclear risk for patient selection, index test, reference standard, flow, and timing for individual studies. An add-on analysis on applicability concerns is also included.

The risk of bias analysis was assessed using the QUADAS-2 tool [11]. The overall risk of bias was high for the included studies (Figure 4). Although most studies recruited patients prospectively, eight patients were recruited with known prostate cancer retrospectively [14,15,17,18,20,22,27].

Three studies investigated the index tests without the knowledge of histopathology [18,20,21], and five studies investigated the reference test unblinded [17,18,19,20,21]. All but one study stated the time interval between mpMRI and PSMA PET/CT [18]. For applicability concerns of this meta-analysis, all studies had low concern for patient selection, while one study had high concern for the index test [17] and three studies had high concern for the reference standard [17,19,21].

## 4. Discussion

We reported the first meta-analysis comparing the diagnostic accuracy of mpMRI and PSMA for detecting clinically significant cancer in matched-patient cohorts. The meta-analysis showed that PSMA PET/CT might be favourable in identifying patients with csPCa (Figure 2). However, we were unable to confirm if this modality is superior in identifying suspected csPCa lesions (Figure 3). The results should be interpreted with the large heterogeneity observed in our study.

The performance of mpMRI and PSMA PET/CT in the per-patient analysis was comparable with the existing literature investigating individual imaging modalities in diagnosing PCa. Zhen et al. investigated the pooled diagnostic accuracy for mpMRI from 29 studies, reporting a good sensitivity of 0.87 (95% CI: 0.81–0.91) and a moderate specificity of 0.68 (95% CI: 0.56–0.79) [28]. Satapathy et al. reported the pooled diagnostic accuracy of PSMA PET/CT from seven studies, with a favourable sensitivity of 0.97 (95% CI: 0.90–0.99) and a moderate specificity of 0.66 (95% CI: 0.52–0.78) [2]. Our meta-analysis revealed a comparable sensitivity of mpMRI (0.87) and slightly reduced sensitivity in PSMA PET/CT (0.93) (Figure 2). The difference may be because our meta-analysis focused on the diagnosis of csPCa as opposed to the diagnosis of PCa in both Zhen et al. and Satapathy et al. [2,28]. However, the specificity for both modalities was remarkably reduced in our analysis compared with the existing literature (mpMRI: 0.47 vs. 0.68; PSMA PET/CT: 0.54 vs. 0.66). The PRECISION trial showed an inverse association of negative MRI-targeted biopsies with lesion conspicuity reported by the PI-RADS v2.0 criteria [29]. Starvrinides and colleagues attempted to capture the characteristics of false-positive MRI lesions, which are distinct from clinically significant diseases [30]. The authors highlighted the use of MRI-calculated PSA density (PSAD) and apparent diffusion coefficient (ADC) as potential predictors of significant disease, a finding verified by the literature [30,31,32]. Although ADC was incorporated in the PI-RADS v2.0 criteria, benign diseases such as prostatitis and prostatic atrophy are known to decrease the signal on the ADC map. Moreover, PSMA expression in non-cancerous prostatic conditions such as inflammation and benign tumours may explain the false-positive cases of PSMA PET/CT as observed in the per-patient analysis [21,33]. Comprehensive interpretations of imaging findings with patient-specific variables, including PSAD and clinical histories, may aid in the distinction of lesions that are likely to be PCa [33]. In our review, PSAD was reported in three studies, while the clinical histories of the included patients were absent from the included studies [17,18,20]. Future studies should include the PSAD and the diagnosis of other prostatic diseases to reduce the diagnosis of false-positive cases.

The purpose of conducting per-lesion analysis was to assess the multifocality of patients with PCa; however, the pooled sensitivity in per-lesion analysis for both the mpMRI and PSMA-PET/CT were unsatisfactory in our review (Figure 3). Previous literature has shown the drawback of segmenting the prostate into sextants. This may lead to the inappropriate assignment of tumour foci on boundaries between sextants, thereby reducing the diagnostic performance compared to only examining targeted histopathological findings [25,34]. In our review, three studies in the per-lesion analysis segmented the specimen into different segments, which may have contributed to the limited diagnostic performance [15,22,23]. In addition, Rhee et al. and Berger et al. conducted their studies when the use of PSMA PET/CT in the diagnosis of prostate cancer was relatively new and lacked the reporting guidelines to make accurate diagnoses [14,22]. Both studies investigated the diagnostic performance from a small patient sample, thereby limiting the generalisability of the results [14,22]. Heterogeneity remained high for both the sensitivity and specificity results, which limits the ability of our study to detect differences between these techniques. Multifocality is a common feature of prostate cancer, as more than one distinct tumour nodule may be present within a prostate gland [22,35]. Although secondary lesions may present with a smaller volume than the index lesions, recent studies suggest that the volume of a tumour may not indicate the biological significance, and smaller tumours may be of greater clinical significance in their impact on prognosis [35]. The per-lesion diagnostic accuracy of PSMA PET/CT has been widely discussed in the context of the staging of prostate cancers [36,37]. However, in the context of primary prostate cancer diagnosis, the existing literature focused on the diagnostic accuracy of per-patient analysis [2]. The accurate localisation of prostate cancer lesions is critical to accurate biopsy and treatment planning [29,38,39]. The low sensitivity for both mpMRI and PSMA PET/CT reported in our study may highlight the need for methods that improve the ability of these techniques to define a lesion’s specific location.

It is noteworthy that the histological criteria for csPCa varied between studies, which may affect the reporting of clinically significant cases and, therefore, the sensitivities and specificities of both modalities. This may also contribute to the high inter-study heterogeneities observed at both the patient and lesion levels. Although GS 7 remains the most common histopathological definition for csPCa [16,17,18,21,22,23], the existing literature has highlighted the clinical significance of the GS 3 + 4 and GS 4 + 3 groups. The GS 4 + 3 groups have a worse prognosis than their counterparts in terms of risk of progression, metastasis, and survival [40,41]. The ISUP grading system has attempted to address the clinical discrepancy between GS 3 + 4 (ISUP 2) and GS 4 + 3 (ISUP 3) by differentiating the two groups in their scoring [42]. However, given the low representation of pattern 4 in <5% of overall tumour volume and the recent introduction of ISUP, it is recommended to report both ISUP grading and GS in the current reporting of prostate cancer [42,43]. The use of a stricter diagnostic criterion for clinically significant prostate tumours may lead to larger estimates of false positives for both mpMRI and PSMA PET/CT. Kalapara et al. showed a lower specificity than other studies in the per-patient analysis [20]. The authors defined clinically significant disease as having ISUP grades of 3–5, which was higher than other studies adopting the ISUP grading system [19]. Future studies, therefore, may be improved through a more standardised classification of csPCa.

Factors affecting the interpretation of mpMRI and PSMA PET/CT images may warrant further investigation. For mpMRI, most included studies commonly used a PI-RADS score of >3 as being potentially indicative of csPCa, but one study in our analysis highlighted that 11 out of 15 patients with PI-RADS 2 scores were proven to have csPCa following histopathological assessment [18]. This may prompt further insight into the more modern PI-RADS v2.0 and v2.1 scoring systems in reducing false-negative reports. It is known that inter-reader variability and reader experience in assigning PI-RADS scores and the subsequent detection of csPCa has been shown to affect the detection rates of csPCa [44,45,46]. However, image quality has also been shown to significantly affect the diagnostic performance of mpMRI [47,48]. Future studies may provide information on image quality using metrics such as the Prostate Imaging Quality (PI-QUAL) score [49].

In PSMA PET/CT analysis, the discrepancies in scoring criteria were more apparent in the clinical suspicion of csPCa. It was not apparent, from the retrieved studies, what the effect is of different volumes of tracer being used and how this may affect imaging interpretation or scores, such as the measurement of SUVmax and MI-ES scores [14,16]. These scoring systems may require further studies concerning a suitable threshold for detecting clinically significant prostate cancer at primary diagnosis, and inter-observer variability within this context [50]. Differences in the PSMA PET/CT scoring criteria may also have contributed to the high observed inter-study heterogeneity within our study, which warrants further analysis in the future. Similar to mpMRI, imaging quality and reader experience have been reported to impact interpretation and diagnostic performance [51,52]. A recent guideline proposed the E-PSMA criteria, which showed lower inter-observer variability and may contribute to the future standardisation of PSMA PET/CT scoring [51].

Studies comparing mpMRI and PSMA PET/MRI were excluded from this meta-analysis owing to differences in PET/MRI methodologies observed in the literature. For example, two studies conducted PET/MRI scans using a hybrid imaging system whereby both MRI and PET imaging acquisition were acquired, simultaneously, in one setting [24,25]. Another two studies defined PSMA PET/MRI as a combination of imaging analyses for mpMRI and PET/CT imaging whereby the images were acquired separately [16,26]. Due to the intrinsic differences in PSMA PET/MRI methods, the number of articles for each PSMA PET/MRI method was insufficient to conduct a meta-analysis. The performance of hybrid PSMA PET/MRI in primary diagnosis has been reported previously [37]. The pooled sensitivity and specificity for the per-patient and per-lesion analyses were 61.5% and 90.9%, and 94.9% and 62.5%, respectively [37]. However, the study did not investigate the performance of combined mpMRI and PSMA PET/CT imaging analysis, which may warrant further systematic review. Moreover, as the study aimed to investigate the performance of PSMA PET/MRI solely. Further diagnostic test accuracy analysis comparing mpMRI alone, PSMA PET alone, and PSMA PET/MRI may be useful to evaluate the optimum imaging technique for the diagnosis of primary csPCa. The aforementioned study was particularly interesting as mpMRI showed high sensitivity in the per-patient analysis and high specificity in the per-lesion analysis. This result was contrary to the reported strengths of PSMA PET/MRI [37].

Genetic factors contributing to mpMRI- or PSMA PET/CT-visible and -invisible prostate cancers may explain the results in our study [12,33]. Visible mpMRI tumours are associated with increased Decipher and Oncotype scores and a greater frequency of phosphatase and tensin homologue (PTEN) loss; no comparable genetic evidence of increased aggression in mpMRI-invisible tumours has been reported [12]. However, genes involving cell structure (such as the actin filament-based process and cytoskeleton organisation) were downregulated in mpMRI-invisible tumours and associated with lower tissue density [12,27,53]. The findings may explain the misdiagnosis of low-cellularity prostate cancer, as mpMRI primarily investigates water molecule content and movement in cancerous prostate cells with high cellularity and contributed to limited sensitivity in our meta-analysis (Figure 2 and Figure 3) [54]. Further validation trials such as the ReIMAGINE Trail (NCT04063566) may depict the role of genetic biomarkers in the use of mpMRI for prostate cancer diagnosis. In contrast to mpMRI, PSMA PET/CT imaging is not dependent on the degree of cellularity, but instead, on the expression of PSMA ligands [33]. It remains to be seen whether PSMA PET/CT also identifies the most high-risk lesions.

Furthermore, the apical expression of PSMA is markedly increased in PCa cells compared with non-cancerous cells [33]. This may require an alternative imaging modality to detect mpMRI-invisible tumours, as PSMA ligand expression is associated with the FOLH1 gene, which is a separate genetic pathway to cell structure expression [12,27,53,55,56]. PSMA induces the activation of phosphoinositide 3-kinase (PI3K) independently of PTEN loss, which contributes to the proliferation of prostate cancer [56]. However, the association between PSMA expression and cellularity remains unknown, currently. Therefore, the combination of PSMA PET/CT and mpMRI imaging for mpMRI-invisible tumours, identified via pre-imaging genetic risk stratification, may be appropriate regarding overall diagnostic accuracy and cost-effectiveness. Previous studies have demonstrated high sensitivity and specificity in combination imaging approaches [16,26]. However, the studies did not categorise patients according to mpMRI-visible and mpMRI-invisible PCa, as all patients were examined using uniform imaging methodologies. Therefore, the additional diagnostic value of PSMA PET when a lesion is mpMRI-invisible may warrant further investigation.

There were several limitations to this systematic review and meta-analysis. First, the limited number of studies investigating the paired analysis between mpMRI and PSMA PET/CT may hinder the robustness of our results. Second, many studies used different definitions of csPCa, and it was impossible to conduct subgroup analysis based on these csPCa definitions to determine their effect. This may explain the high heterogeneity amongst included studies. Third, the insignificant statistical result in the per-patient analysis may require further investigation to determine if any differences exist in the specificity of mpMRI and PSMA PET/CT. Our meta-analysis was unable to be conducted for PSMA PET/MRI fusion techniques owing to the different definitions for PET/MRI fusion.

Nevertheless, our study represents the first meta-analysis of the diagnostic accuracy of mpMRI and PSMA PET/CT on primary prostate cancer. It is based on a robust research methodology with strict criteria for study selection. Given the current limitations, further research should continue to contribute to the evidence base and address the heterogeneity observed in the study. Future research should standardise the interpretation of PSMA PET/CT images and histopathology scoring systems to address the methodological discrepancies.

## 5. Conclusions

This meta-analysis shows that, at the per-patient level, PSMA PET/CT may perform better than mpMRI in detecting primary prostate cancer. In contrast, both modalities were comparable in locating specific lesions in patients. PSMA PET/CT is a whole-body procedure and may add intrinsic value compared to pelvic mpMRI. However, considerable heterogeneity was observed in our study. Therefore, there is a need to standardise imaging interpretation and histopathology scoring systems to reduce variation between studies. Further analyses should focus on the diagnostic performance of combined mpMRI and PSMA PET/CT imaging modalities.

## Figures and Tables

**Table 1:** Overview of studies included for both per-patient and per-lesion analysis.

Authors	Year	Ref	No. of Patients	No. of Lesions	MRI Imaging Power	PI-RADS Version	mpMRI Positivity Criteria	PSMA PET Scoring System	PSMA Tracer	PSMA Tracer Injected (MBq)	PSMA PET/CT Positivity Criteria	Mean Age (yrs)	Mean PSA Value (ng/dL)	Reference Standard	Clinically Significant Definition
**Berger**	2018	[14]	50	84	3T	v2.0	PI-RAD ≥ 3	SUVmax	68Ga-PSMA-11	-	SUVmax > 2.5	64.9 (59.3–70.5)	10.6 (2.5–18.7)	WMP	ISUP ≥ 1
**Bettermann**	2019	[15]	17	193 (772 quadrants)	3T/1.5T	v2.0	PI-RADS ≥ 3	Uptake against background	68Ga-PSMA-11	172 (138–206)	Uptake superior to the background activity in >1 slice	67 (48–76)	17.4 (6.01–218.0)	WMP	Lesions extending > 3 mm into another quadrant
**Chen**	2019	[16]	54	90	3T	v2.0	PI-RADS ≥ 4	MI-ES Score	68Ga-PSMA-11	131.7 (130.6–177.6)	MI-ES ≥ 2	69 (55–84)	13.53 (4.04–110.00)	WMP	Cancer volume ≥ 0.5 cm^3^/GS ≥ 3 + 4 /Stage ≥ pT3
**Donato 2019**	2019	[17]	58	88	3T	v2.0	PI-RADS ≥ 3	3-point Likert Scale ^a^	68Ga-PSMA-11	150.0 (142.5–157.5)	SUVmax > 5 (Equivocal)	65.5 (60–68)	7.35 (5.6–12)	WMP	GS ≥ 3 + 4
**Donato 2020**	2020	[18]	144	-	3T	v2.0	PI-RADS ≥ 3	3-point Likert Scale ^a^	68Ga-PSMA-11	150.0 (142.5–157.5)	>Equivocal	66.5 (61.7–71.25)	8.6 (6–12.25)	Ultrasound-guided transperineal targeted biopsies	GS ≥ 3 + 4
**Emmett**	2021	[19]	291	-	3T/1.5T	v2.0	PI-RADS ≥ 3	4-point Certainty Scale ^b^	68Ga-PSMA-11	1.8–2.2 MBq/kg	Positive (Probably/Definite)	64.0 (58.7–69.9)	5.6 (4.2–7.5)	Systematic transperineal biopsies	ISUP ≥ 2
**Kalapara**	2020	[20]	205	-	3T	v2.0	PI-RAD ≥ 3	Binary Scale	68Ga-PSMA-11	1.8–2.2 MBq/kg	Lesion with the highest avidity by SUVmax	67 (61–72)	7.18 (4.90–10.20)	WMP	ISUP ≥ 3
**Li**	2020	[21]	67	-	3T	v2.0	PI-RAD ≥ 3	Uptake against background	68Ga-PSMA-617	111–185	Uptake superior to the background activity	68 (42–85)	10.48 (3.15–19.76)	Transrectal ultrasound biopsy	GS ≥ 7
**Rhee**	2016	[22]	22	71 (540 segments)	3T	v1.0	PI-RAD ≥ 3	Uptake against background	68Ga-PSMA-11	150	Uptake superior to the background activity	62 (55–69)	6.1 (0–14.6)	WMP	GS ≥ 4 + 3 +/− tumour size ≥ 6 mm
**Zamboglou**	2021	[23]	10	14 (601 segments) *	3T	v2.1	PI-RAD ≥ 3	Uptake against background	[18F]PSMA-1007	310 (249–370)	Uptake superior to the background activity	-	-	WMP	GS ≥ 7 *

^a^—likely, equivocal, unlikely; ^b^—definitely negative, probably negative, probably positive, definitely positive; MI-ES—Molecular Imaging PMSA Expression; GS—Gleason score; ISUP—International Society of Urological Pathology; WMP—Whole-mount prostatectomy. * Obtained by contacting the author directly.
